# Mechanisms of Specificity for Hox Factor Activity

**DOI:** 10.3390/jdb4020016

**Published:** 2016-05-09

**Authors:** Arya Zandvakili, Brian Gebelein

**Affiliations:** 1 Molecular and Developmental Biology Graduate Program, Cincinnati Children’s Hospital Medical Center, Cincinnati, OH 45229, USA; 2 Medical-Scientist Training Program, University of Cincinnati College of Medicine, Cincinnati, OH 45267, USA; zandvaaa@mail.uc.edu; 3 Division of Developmental Biology, Cincinnati Children’s Hospital Medical Center, Cincinnati, OH 45229, USA

**Keywords:** Hox, transcription factor, *cis*-regulatory modules, DNA binding specificity

## Abstract

Metazoans encode clusters of paralogous Hox genes that are critical for proper development of the body plan. However, there are a number of unresolved issues regarding how paralogous Hox factors achieve specificity to control distinct cell fates. First, how do Hox paralogs, which have very similar DNA binding preferences *in vitro*, drive different transcriptional programs *in vivo*? Second, the number of potential Hox binding sites within the genome is vast compared to the number of sites bound. Hence, what determines where in the genome Hox factors bind? Third, what determines whether a Hox factor will activate or repress a specific target gene? Here, we review the current evidence that is beginning to shed light onto these questions. In particular, we highlight how cooperative interactions with other transcription factors (especially PBC and HMP proteins) and the sequences of *cis*-regulatory modules provide a basis for the mechanisms of Hox specificity. We conclude by integrating a number of the concepts described throughout the review in a case study of a highly interrogated Drosophila *cis*-regulatory module named “The Distal-less Conserved Regulatory Element” (DCRE).

## 1. Introduction

Hox proteins are a family of homeodomain-containing Transcription Factors (TFs) that are critical for regulating developmental processes in metazoans [1,2]. The stark homeotic changes occurring in Hox mutant animals, such as the classic antenna-to-leg transformations in *Drosophila*, testify to the importance of Hox factors for proper development [3]. In addition, more recent studies have demonstrated that Hox factors not only drive stereotypic developmental programs, but also have a role in maintaining differentiated cell populations [4,5]. Given that Hox factors are conserved from *C. elegans* to *H. sapiens*, a fundamental understanding of how Hox factors function will yield significant insights into both the development and evolution of body plans.

**Figure 1:**
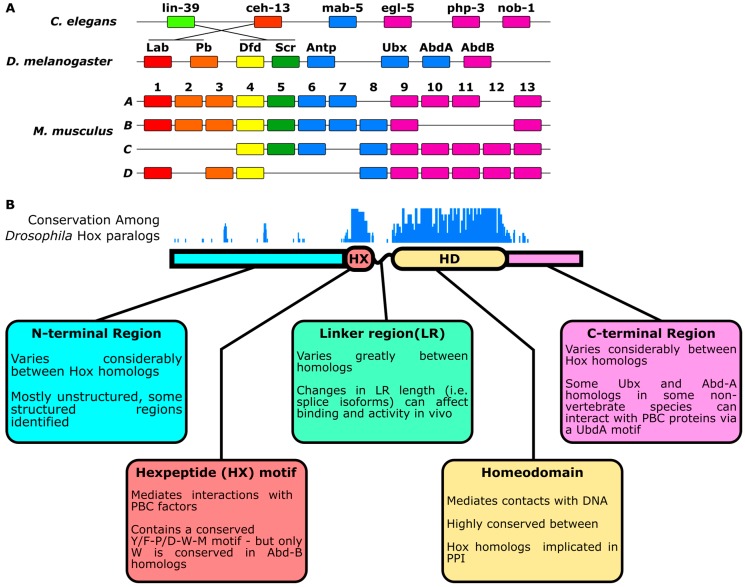
Schematic of Hox gene locus and Hox proteins. (**A**) Schematic of *Hox* gene clusters in *C. elegans*, *D. melanogaster* and *M. musculus*. Genes with similar colors are thought to derive from a common ancestor [10]; (**B**) Schematic of a Hox protein with regions labeled and described in boxes (bottom) [11,12,13,14,15,16,17,18]. Per-amino-acid conservation score across *Drosophila* Hox protein sequences demonstrates that the HX and homeodomain regions are the most conserved regions across paralogous Hox proteins. Multiple sequence alignment was produced via ClustalΩ (top) [19,20]. Note: The size of each Hox protein region is not to scale.

The majority of metazoan genomes encode clusters of paralogous Hox genes (Figure 1A). While invertebrates generally have one Hox cluster, vertebrates have multiple Hox clusters owing to duplications of the entire cluster during evolution [6]. The order of Hox genes within the clusters typically correlates with their expression pattern along the Anterior-Posterior (A-P) axis of the embryo [7,8,9]. Genes at the 3′ end of a cluster are expressed in anterior regions of an embryo, whereas genes toward the 5′ end are expressed in progressively more posterior regions of the embryo. Thus, the differential expression of Hox genes is a key step in the specification of distinct cell fates along the A-P axis.

At the sequence level, Hox proteins share two stereotypic domains: a conserved homeodomain and a conserved Hexapeptide (HX) motif that is located N-terminal to the homeodomain (Figure 1B). Hox factors utilize their homeodomains to directly bind DNA, and they share very similar binding preferences *in vitro* as monomers [21,22,23]. The HX motif mediates direct interactions with another family of TFs (PBC proteins), and it is separated from the homeodomain by a flexible and highly variable linker region [12,13]. Outside of the homeodomain and the HX motif, Hox protein sequences diverge substantially; and while we largely do not understand their functions, these non-conserved regions contain residues that can be post-translationally modified and/or have been implicated in protein-protein interactions and the regulation of transcriptional outputs [24,25,26,27].

Hox factors specify cell-fates based on their ability to interact with *Cis*-Regulatory Modules (CRMs) and to regulate transcription [28,29]. CRMs provide a DNA platform to organize interactions between Hox factors and other proteins. Although Hox interactions with DNA and other proteins have been studied extensively, we still lack a comprehensive understanding of how specificity is achieved to accurately recognize and regulate target genes required to direct specific cell fates. In this review, we explore three questions related to Hox specificity: First, how do paralogous Hox proteins drive different cell fates even though they utilize conserved homeodomains with highly similar DNA binding preferences? Second, metazoan genomes contain a large number of potential Hox binding sites, yet only a subset of these sites are bound at any one time. What factors determine which sequences are bound and regulated by Hox proteins, whereas other sequences containing Hox binding sites remain unbound? Third, once bound, how does an individual Hox factor activate some target genes and repress others? Below, we present emerging evidence that provides new insight into the mechanisms that contribute to the specificity of Hox action.

## 2. Differentiation of Hox Paralog Activities

A fundamental problem in the study of TF specificity is that most TFs are members of large protein families that have highly similar DNA binding properties yet distinct *in vivo* functions [30]. The Hox family of TFs is an exemplar of this problem. While paralogous Hox factors bind highly similar DNA sequences, genetic loss- and gain-of-function studies demonstrate that Hox factors control diverse cell fates along the A-P axis of metazoans [1,31]. Furthermore, in *C. elegans*, it has been demonstrated that paralogous Hox proteins have highly divergent genomic binding patterns *in vivo* [32]. However, it is not immediately clear whether this difference in *in vivo* function is due to differences in Hox paralogs or due to the fact that Hox paralogs are functioning in different cellular contexts.

Several studies have controlled for cellular context and have demonstrated that paralogous Hox proteins have different *in vivo* activities within the same cell types. First, over- or under-expressing specific Hox paralogs within the same cell types can result in different phenotypes [33,34,35]. For instance, in *C. elegans,* when the Hox genes *egl-5* and *lin-39* are expressed under the control of regulatory elements from the *mab-5* locus (a different Hox gene), they do not rescue the *mab-5* mutant phenotype [35]. Second, the misexpression of different Hox genes results in distinct changes in global gene expression patterns. For example, six *Drosophila* Hox genes were individually expressed in a ubiquitous pattern with the same Gal4 driver line in *Drosophila* embryos, and RNA was isolated to compare changes in gene expression [36]. Of the genes that changed in expression, the majority (nearly 70%) changed in response to a single Hox factor, while only 1.3% of the genes changed in response to all six Hox factors [36]. Third, a growing number of Hox-regulated CRMs has been identified, and many are regulated by only one or a small subset of paralogous Hox factors when tested in the same cellular contexts [37,38,39,40,41]. While these studies all support the notion that Hox factors largely control distinct cell fates, it has been found that, at least in some contexts, Hox factors can produce very similar phenotypes when expressed in the same cell types. For example, in *Drosophila*, all Hox factors (except abdominal-B) can substitute for Labial (Lab) in specifying tritocerebral neuromere [42]. Furthermore, in vertebrates, Hox mutant phenotypes become more severe when multiple Hox genes with overlapping expression are mutated, suggesting that Hox genes have some functional redundancy [9,43]. Thus, each Hox factor is likely to regulate both common and paralog-specific target genes and cell fates *in vivo*.

**Figure 2:**
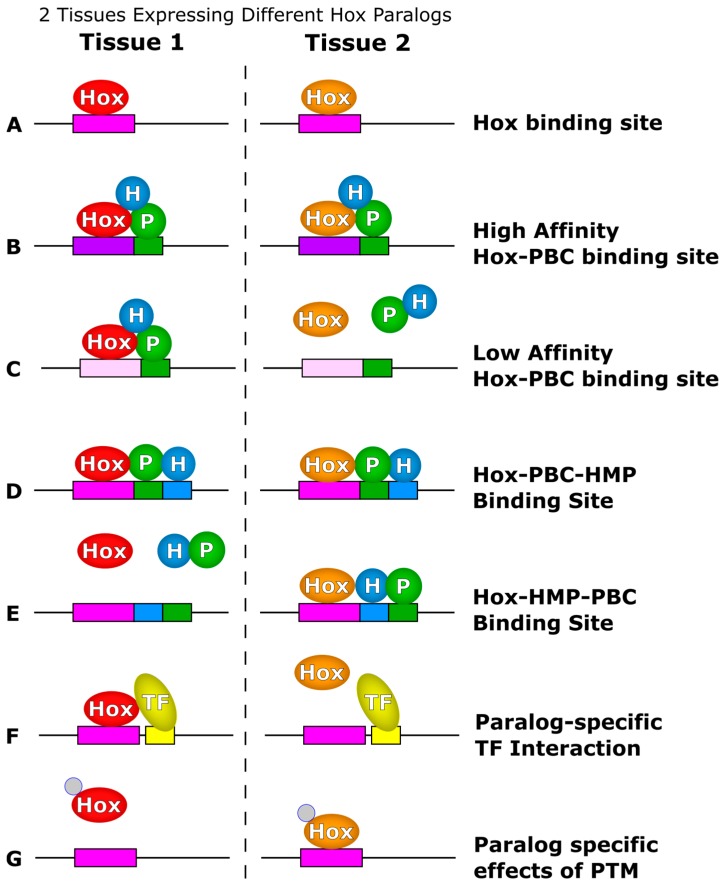
Summary of the mechanisms of Hox paralog specificity. (**A**) A single Hox site is unlikely to differentiate between two different Hox paralogs; (**B**,**C**) Low-affinity Hox-PBC binding sites are more likely to differentiate between Hox paralogs than high affinity Hox-PBC binding sites [23,41]; (**D**,**E**) All Hox paralogs can typically bind adjacent Hox-PBC sites, but adjacent Hox-HMP sites have a preference for posterior Hox paralogs [26,44,45]; (**F**) Interactions with a nearby binding site for another TF can cause a CRM to be paralog specific [46,47]; (**G**) The same post-translational modifications (gray circle) can affect paralogous Hox proteins differently [48,49,50,51]. “P” (PBC protein) and “H” (HMP protein).

Here, we focus on how the cooperation with other sequence-specific TFs aids in the differentiation of the binding preferences and activities of Hox paralogs. We describe how interactions between Hox factors and the PBC family of homeodomain proteins uncover the importance of latent DNA binding specificity and low affinity binding sites in generating Hox-specific outputs. We subsequently explore how the HMP family of homeodomain proteins selectively interacts with posterior Hox factors. In addition, HMP TFs also directly interact with the PBC proteins and thereby form higher-order TF complexes with Hox factors that can enhance both DNA binding affinity and specificity. Next, we provide a few examples of the diversity of other TFs that interact with subsets of Hox factors, either directly or via nearby binding sites encoded within CRMs. Finally, we describe how post-translational modifications differentially affect the activities of paralogous Hox proteins. A summary of these mechanisms is provided in Figure 2.

### 2.1. Interactions with PBC Factors Reveal Hox Latent Specificity

**Figure 3:**
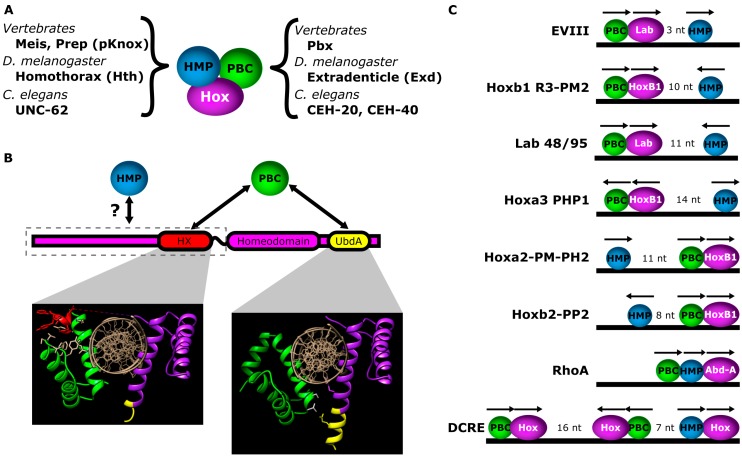
Interaction between Hox factors and PBC/HMP proteins. (**A**) Names of PBC and HMP homeodomain proteins in *C. elegans*, *Drosophila* and vertebrates; (**B**) Motifs in Hox factors used to mediate interactions with PBC and HMP proteins (top). Yeast-2-hybrid data suggest sequences N-terminal to the homeodomain mediate interactions with homothorax [26]. Hox proteins can mediate interactions with PBC proteins via the HX motif or, in the case of non-vertebrate Abd-A and Ubx homologs, the UbdA motif. *Structural panels*: Structures produced by Foos *et al.*, 2015, demonstrating both HX and UbdA interaction modes between *Drosophila* Ubx and Exd. PBC protein in green; Ubx protein in purple; HX motif in red; UbdA motif in yellow [18]. When bound to a canonical Hox-PBC binding site, Hox and PBC proteins bind on opposite sides of the DNA. *Left structural panel*: The HX motif (red) mediates interactions with a hydrophobic pocket formed by helix 1, helix 3 and the TALE motif of the Exd homeodomain. *Right structural panel*: The UbdA motif mediates interactions with helix 3 of the PBC homeodomain. UbdA can either be unstructured or form an α-helical extension of the third helix of the Hox homeodomain. Note: The Ubx protein fragment in the left panel contains both the HX and UbdA motifs, while the Ubx protein fragment in the right panel only contains the UbdA motif. (**C**) Examples of CRMs with Hox-PBC-HMP binding sites, demonstrating variations in the order, orientations and spacing of the binding sites. The space between HMP and Hox-PBC sites is indicated. EVIII [64]; Lab 48/95 [65]; R3-PM2 [66]; PP2 [67]; PM-PH2 [68]; PHP1 [69]; RhoA [40]; DCRE [70].

First identified in 1990, PBC proteins were nearly simultaneously discovered as modulators of Hox function in *Drosophila* and proto-oncogenes in mammals [52,53,54]. The PBC family includes Extradenticle (Exd) in *Drosophila*, CEH-20 and CEH-40 in *C. elegans* and Pbx factors in mammals; and all PBC proteins contain a highly-conserved homeodomain that differs from a canonical homeodomain by the addition of a Three Amino-acid Loop Extension (TALE) motif between helix 1 and helix 2 of the homeodomain [55] (Figure 3A). It was recognized early on that Hox and PBC proteins bind DNA cooperatively [56,57,58] and that PBC proteins were essential for Hox function [59,60,61]. Subsequent structural studies determined that Hox-PBC interactions on DNA were mediated via insertion of the Hox HX motif into a hydrophobic pocket of the PBC homeodomain that is composed of residues from the TALE motif, helix 1 and helix 3 [12,13] (Figure 3B). The Hox HX motif is located N-terminal to the homeodomain and contains a highly-conserved Y/F-P/D-W-M sequence (Figure 1B), where the W residue is critical for making hydrophobic interactions with the PBC TALE motif [12]. While the majority of Hox factors have a defined HX motif, the posterior Abdominal-B (Abd-B) or Hox paralog group 9–13 factors only rely upon a conserved W residue to mediate this interaction [62]. Importantly, the interaction between Hox and PBC factors occurs through nearby DNA binding sites for each factor and, thereby, results in both enhanced DNA binding specificity and affinity [23,56,63].

In addition to enhancing DNA binding affinity, interactions between TFs can result in changes in binding preferences via allosteric changes in conformation of the TFs and/or the DNA [71,72,73]. The concept of revealing differences in binding preferences between similar TFs is known as latent specificity. The latent specificity mechanism is well-established for interactions between Hox and PBC proteins. Soon after the discovery of PBC proteins, biochemical evidence demonstrated that PBC proteins modify the DNA binding specificity of *Drosophila* and mammalian Hox paralogs [56,57,74,75]. This concept was most comprehensively tested *in vitro* by a 2011 SELEX-seq (Systematic Evolution of Ligands by EXponential enrichment followed by sequencing) study, which demonstrated that *Drosophila* Exd-Hox heterodimers have a greater diversity of binding site preferences than Hox monomers [23]. Specifically, it was found that interactions between Hox factors and Exd result in the selection of distinct sequences within six core nucleotides (A_5_YNNAY_10_) of the Hox binding site (note, the combined PBC-Hox site typically consists of 12 nucleotides, nnTGAYnnAYnn). Interestingly, the binding preference for Exd-Hox dimers segregated with the expression of Hox factors along the A-P axis; thus, the preference for a core motif can be grouped into distinct clusters of anterior (Labial and Proboscipedia), middle (Deformed and Sex combs reduced) and posterior Hox factors (Antennapedia, Ultrabithorax, Abdominal-A and Abdominal-B). These preferences also segregated with the predicted width of the DNA minor groove within the Exd-Hox core-motif. Specifically, it was found that anterior Hox factors select sequences with a narrow minor groove in position A_9_Y_10_, while posterior Hox factors select sequences with wider minor grooves [23,76]. In addition to differentiating preferences within the Exd-Hox core-motif, there were distinct preferences between Hox factors for sequences flanking the Hox half of the Exd-Hox motif, especially for anterior Hox factors [23]. However, there is an exception to the rule of Exd differentiating preferences; interaction with Exd actually made the preferences of Ultrabithorax (Ubx) and Abd-B more similar [23].

Structural studies have revealed that interactions with Exd on DNA allow protein sequences that are more variable between Hox paralogs (namely the linker region and N-terminal arm of the homeodomain) to make a greater contribution to DNA binding. Comparing crystal structures of Exd and the Sex Combs Reduced (Scr) Hox factor bound to either a selective sequence specifically bound by Scr (*fkh250*) or a non-selective consensus Exd-Hox site bound by many Hox factors (*fkh250con*) revealed significant differences in the mechanism of DNA binding [71]. On the selective sequence, the Scr N-terminal arm is structured and inserted into the minor groove, whereas these same amino acids are disordered and make a minimal contribution to Scr binding on the *fkh250con* sequence [71]. Selectivity was found to depend upon two residues—Histidine-12 (His-12, in the Linker Region) and Arginine 3 (Arg3, in the N-terminal arm)—that insert into the minor groove of *fkh250*. His-12 and Arg3 are highly conserved among Scr homologs, suggesting that these residues are important to Scr function. Since His-12 and Arg3 do not make direct hydrogen bonds with bases, it was proposed that these two residues recognize the shape of the DNA. Indeed, a recent SELEX-seq study using Hox-Exd dimers demonstrated that mutating His-12 and Arg3 in Scr results in less selectivity for sequences that are predicted to have narrow minor grooves at position A_8_Y_9_ [76]. Furthermore, mutating the linker region and the N-terminal arm of the homeodomain in Antennapedia (Antp) to match those of Scr not only changed Exd-Antp binding preferences to be very similar to those of Exd-Scr, but it also allowed the mutant Antp protein to activate the Scr-specific enhancer (*fkh250*) *in vivo* [76]. This SELEX-seq study is also notable for demonstrating that using both DNA shape-readout, as well as DNA base-readout greatly improves the predictions of sequence-specific affinity of Hox-Exd complexes.

Importantly, the concept of latent specificity may be broadly applicable to the study of TFs in general. A recent large-scale SELEX-based screen of cooperative DNA binding between TF pairs identified additional examples of latent specificity. For instance, HoxB2 was found to diversify the binding preferences of a subset of ETS factors (E-26 transcription factors) [45]. Interestingly, this screen demonstrated that of the 315 TF-TF heterodimers that were analyzed, 2/3 of the heterodimeric binding preferences were substantially different than the monomeric binding preferences of the individual TFs. However, in many cases, when a set of paralogous TFs interacted with the same partner, the resultant binding preference was very similar, suggesting that latent specificity is unlikely to be the sole source of distinguishing the activities of paralogous TFs.

### 2.2. Low-Affinity Binding Sites Distinguish Hox-PBC Dimers

The latent specificity mechanism demonstrates that adjacent Hox-PBC binding sites in CRMs can contribute to paralog-specific binding of CRMs. However, many of the highest affinity sites identified from these assays can be bound by several paralogous Hox factors, suggesting that high-affinity sites may be regulated by many Hox factors. More recently, a study proposed that CRMs distinguish between Hox paralogs via low-affinity binding sites [41]. The idea behind this proposal is that TFs within a family have very similar DNA binding domains that vary only in sites that make weak interactions with DNA. Therefore, the variations in DNA-binding domains only make minor contributions to the overall binding energy when binding high affinity sites. By contrast, subtle differences in DNA binding domains can make a larger contribution to the overall binding energy when interacting with low-affinity sites. The binding of the *fkh250* sequence by Scr described above is an example of how a lower affinity site depends on Scr-specific amino acid contacts for DNA binding, whereas a higher affinity site (*fkh250con*) only requires homeodomain amino acids that are conserved in many Hox paralogs [71]. In addition, Crocker *et al*. identified a cluster of low-affinity Ubx-Exd binding sites within enhancers in the *Drosophila* *shavenbaby* locus, and they found that replacing these sites with higher affinity Hox-Exd sites allowed the activation of the enhancer by other Hox factors in reporter assays [41]. Interestingly, it is not uncommon to find functional, low-affinity binding sites within developmentally-important enhancers [41,77,78,79,80], suggesting that low affinity might provide a selective advantage over high-affinity binding sites. However, the prevalence of low-affinity binding sites in developmentally-important enhancers may also be a function of the fact that for any given TF, a greater number of sequences can yield low-affinity binding sites than high affinity binding sites. Crocker *et al*. provide a recent review of low-affinity binding sites and their potential functions [81].

### 2.3. Additional PBC Interaction Surfaces Differentiate Hox Paralogs

While it has been widely demonstrated that the Hox HX motif mediates Hox-PBC interactions, results from several biochemical experiments suggest that it is not the sole mechanism for Hox-PBC interactions. In fact, mutating or removing the HX motif in Lab, Ubx or Abdominal-A (Abd-A) does not abolish the ability of these Hox factors to interact with Exd on DNA [15,56,82,83,84,85,86,87]. Furthermore, *in vivo* Exd-dependent activities for Lab, Ubx and Abd-A were largely not abolished by HX mutations [15,57,83,84,85,86,87]. In addition, recent *in vivo* fluorescence complementation assays (Bimolecular Fluorescence (BiFC)) have demonstrated that the majority of Hox factors are not completely dependent on the HX motif to form complexes with Exd [88]. These findings indicate that Hox factors can use alternative mechanisms to interact with PBC factors to regulate gene expression.

An alternative PBC interaction motif has been identified in the *Drosophila* Ubx and Abd-A Hox factors. This motif is conserved among arthropod and cnidarian Ubx and Abd-A homologs and, hence, has been named the UbdA motif [18]. The UbdA motif is located immediately C-terminal to the homeodomain. Biochemical and *in vivo* BiFC assays have demonstrated that the UbdA motif contributes to interactions with Exd [15,84,86,88]. Moreover, mutation of the UbdA motif in Abd-A and Ubx results in decreased Exd-dependent activities *in vivo* [15,18,84,86]. More recently, a crystal structure of Ubx interacting with Exd via its UbdA domain has been described. Foos *et al*. crystalized two types of Ubx/Exd/DNA complexes, one using Ubx proteins with both the HX and UbdA motif and one with only the UbdA motif [18]. In both complexes, the homeodomains of Ubx and Exd were situated on opposite sides of the DNA. When both the HX and UbdA motifs were present in Ubx, only the HX motif made contacts Exd, whereas the UbdA motif was mostly disordered. By contrast, if only the UbdA only is present in Ubx, the UbdA forms a coiled extension off of the homeodomain’s third helix and makes contact with the loop region between the second and third helices of the Exd homeodomain (Figure 3B). The UbdA-Exd interaction occurs on the opposite side of the DNA relative to the HX-Exd interaction. Based on these structures, it is not clear what regulates whether the HX and/or UbdA mediates interactions between Ubx/Abd-A and PBC proteins. However, Saadaoui *et al*. (2011) demonstrated that Ubx isoforms with longer linker region lengths are more resistant to HX mutations in regulating a CRM containing adjacent Exd-Ubx binding sites [15]. Given the topology, this may be due to the fact that the longer linker region length gives flexibility to Ubx and Abd-A proteins to mediate contacts on both sides of the DNA via the HX and UbdA motifs.

### 2.4. Interactions with HMP Proteins Differentiate Hox Paralogs

In addition to PBC factors, another group of TALE homeodomain proteins directly associates with Hox factors on DNA and contributes to Hox paralog specificity. The HMP family of proteins includes Homothorax (Hth) in *Drosophila*, UNC-62 in *C. elegans* and Meis and Prep (pKnox) in vertebrates. Hth was originally identified as a gene important to larval cuticle development in *Drosophila*, while Meis was originally identified as a proto-oncogene in mammals [89,90]. These genes were later shown to interact with PBC and Hox proteins and thereby contribute to Hox-mediated outcomes [26,44,91].

The vertebrate HMP proteins, Meis1A and 1B, have been shown to interact with Hox factors from multiple paralog groups (murine Hox paralog groups 2, 4, 5, 8, 9, 10, 11, 12 and 13) using yeast two-hybrid assays [26]. However, DNA binding assays indicate that only posterior Hox factors (Abd-B family members in particular) bind cooperatively with HMP proteins to DNA containing adjacent Hox-HMP binding sites [26,44,45]. In *Drosophila*, the Ubx and Abd-A Hox factors have also been shown to directly cooperate with Hth by binding and regulating CRMs containing adjacent Hox-Hth binding sites [40,46]. These findings suggest that the arrangement of adjacent Hox-HMP binding sites may contribute to paralog specificity by favoring posterior Hox factors over anterior ones. However, the crystal structures of Hox-HMP-DNA complexes have not been solved, and therefore, the mechanism and specificity of interaction between Hox and HMP factors remains unclear.

In addition to interacting with Hox factors, the TALE PBC and HMP proteins heterodimerize through highly-conserved domains found N-terminal to their homeodomains. Importantly, PBC-HMP heterodimer formation increases the nuclear import of PBC, which is largely cytoplasmic in the absence of HMP proteins, and enhances the stability of HMP proteins [91,92,93,94,95]. More recently, it has been shown that the Hox-PBC-HMP interactions are ancient and evolutionarily conserved among cnidarians and bilaterians and that Hox, PBC and HMP from different species can interact with one another and substitute for one another in functional assays [96]. Molecularly, Hox-PBC-HMP interactions result in the formation of higher order complexes on DNA [97,98,99,100]. In fact, several CRMs containing binding sites for all three factors have been identified, especially in the vertebrate hindbrain and in *Drosophila* [40,46,58,64,65,66,67,69] (Figure 3C). In general, these CRMs contain either an adjacent PBC/Hox site with a nearby HMP site or an adjacent HMP/Hox site with a nearby PBC site. Analysis of binding site patterns in these CRMs reveals a variety of spacing and orientations of Hox, PBC and HMP binding sites that are sufficient to mediate the formation of Hox/PBC/HMP complexes on DNA [101] (Figure 3C). Moreover, one recent study found that hindbrain enhancers in zebrafish are enriched for HMP binding sites within 50 bp of combined Hox/PBC sites [102]. While this study did not conclusively demonstrate that these CRMs were directly regulated by Hox/PBC/HMP complexes, it does illustrate the potential flexibility of spacing for interacting HMP, Hox and PBC binding sites. In addition, the fact that certain interactions between Hox and PBC/HMP are paralog specific, *i.e.*, the UbdA interaction motif in Ubx/Abd-A, the non-conserved HX motif in Abd-B homologs, the cooperative DNA binding between HMP proteins and posterior Hox factors, suggests the possibility that particular Hox-PBC-HMP binding site arrangements are paralog specific. Future studies focused on analyzing a large number of Hox-regulated CRMs will be needed to determine if specific arrangements of binding sites are an important contributor to Hox paralog specificity.

### 2.5. Hox Paralogs Have Different Partner Preferences

As described for the PBC, HMP and Hox factors, interactions between TFs can yield cooperative complex formation on DNA, and the binding of each TF contributes to the overall binding affinity and specificity. In addition to PBC and HMP proteins, other sequence-specific TFs have been shown to interact with Hox factors, and several of these interactions are paralog specific. Thus, a CRM may encode Hox-paralog-specific activities by including a nearby binding site for a paralog-specific interacting TF.

Large-scale screens have identified additional TFs that interact with specific Hox factors [45,103]. For instance, Baëza *et al.* tested the ability of five *Drosophila* Hox factors (Scr, Antp, Ubx, Abd-A and Abd-B) to interact with 35 other TFs using an *in vivo* BiFC assay [103]. This study demonstrated that each tested Hox factor interacts with a distinct combination of the 35 TFs. On average, there was a 59% pairwise similarity between the sets of TFs that interacted with each Hox factor. Presumably these differences in affinity for other TFs will contribute to the differential binding of Hox factors to genomic sequences. However, there is currently limited data showing that the interactions between the Hox factors and the 35 other TFs occur on DNA, much less affect DNA binding specificity.

The data above suggest that Hox-regulated CRMs will require additional TF binding sites to yield paralog-specific outputs. This model is supported by examples of specific CRMs [40,46]. For instance, a *rhomboid* (*rho*) CRM (*RhoA*) in *Drosophila* contains binding sites for both an abdominal Hox complex composed of Exd/Hth/Abd-A, as well as the Pax2 transcription factor [47]. Importantly, mutations in either the Hox site or the Pax2 site disrupt activation, demonstrating that both the abdominal Hox complex and Pax2 protein need to bind the CRM for proper output. Moreover, the *RhoA* CRM is only activated by one specific abdominal Hox factor (Abd-A) that interacts with Pax2, whereas a thoracic Hox factor, Antp, does not interact with Pax2 and is unable to stimulate this CRM. This difference in ability to form complexes with Pax2 is thought to contribute to the paralog-specific activity of *RhoA*. Moreover, the interaction between specific posterior Hox factors and Pax2 may extend to vertebrates, as well. HoxA11 has been shown to interact with Pax2, and binding sites for both factors are required for the expression of a Six2 CRM in the developing mouse kidney [104].

### 2.6. Post-Translational Modifications Differentially Affect Hox Paralogs

Several different enzymes post-translationally modify Hox factors [48,49,50,51,105,106,107,108,109,110,111]. Here, we review those modifications that are known to differentially affect Hox paralogs.

Poly(ADP)-Ribose Polymerase-1 (PARP-1) was shown to poly(ADP)-ribosylate several mammalian Hox factors (HoxA5, HoxA7, HoxB6, HoxB7, HoxC6, HoxC8), but this modification only reduces the *in vitro* DNA binding and transcriptional activity of HoxA7 and HoxB7 [48]. This study demonstrated that HoxA7 and HoxB7 have a long glutamate-rich repeat in their C-terminal motif that is necessary for poly(ADP)-ribosylation and that the addition of a long glutamate-rich repeat in HoxB6 was sufficient to allow poly(ADP)-ribosylation to disrupt HoxB6 activity *in vitro* [48]. Similarly, it has been shown that phosphorylation by Casein Kinase II (CKII) differentially affects Hox paralogs. Phosphorylation of the HoxA9 homeodomain by CKII decreases DNA binding and thereby diminishes the ability of HoxA9 to keep cultured hematopoietic progenitors from differentiating [49,50]. By contrast, HoxB7 appears to gain activity after phosphorylation. Two CKII phosphorylation sites (S132 and T203) were shown to flank the HoxB7 homeodomain, and mutating these sites to alanine inhibited the ability of HoxB7 to maintain hematopoietic cell culture in an undifferentiated state [51]. However, in both of these cases, it has not yet been delineated what mechanisms signal for the Hox paralogs to be post-translationally modified by these enzymes. An intriguing possibility is that post-translational modifications may occur at CRMs. Previous studies have shown that post-translational modifiers, such as PARP and CKII, can be recruited to CRMs and regulate gene expression [112,113,114], but it is unclear whether post-translational modifications of Hox factors is a CRM-dependent process or a global mechanism of regulating Hox factor activity. If the former is true, recruitment of post-translational modifiers to DNA could potentially contribute to paralog-specific activity of individual CRMs as well.

## 3. Target Accessibility

In order for Hox factors to regulate gene expression, they must gain access to target CRMs. There is substantial evidence that the binding patterns of most TFs are primarily determined by nucleosome positioning [115]. Are Hox factors like most TFs in that they only bind genomic sequences found in open chromatin or do they have pioneer activity capable of interacting with DNA wrapped in nucleosomes? Here, we present recent evidence that the Hox factor binding may be largely constrained to open chromatin and provide examples demonstrating that Hox factors rely on other TFs to open chromatin prior to Hox binding.

If Hox factors significantly alter the genomic chromatin landscape, one would predict that serially homologous tissues that express different Hox factors along the body plan would differ in their chromatin accessibility profiles. In *Drosophila*, the wing and haltere are serially homologous appendages with the wing arising from the second thoracic segment (T2) and the haltere developing from the third thoracic segment (T3) [116]. Previous studies demonstrated that the Hox factor, Ubx, is necessary to specify the haltere. In fact, genetic removal of Ubx can transform the haltere into wing tissue, whereas the misexpression of Ubx in the second thoracic segment transforms the wing into a haltere-like structure [7,117]. Comparisons of genome accessibility between the haltere and wing discs, however, revealed a largely similar chromatin landscape [118]. This finding suggests that the expression of Ubx does not alter cell fates by global changes in chromatin structure, but by acting upon accessible regulatory elements that are present in both the wing and haltere imaginal discs.

As a more direct test of the ability of Hox factors to alter genome accessibility, recent studies in cell culture revealed that Hox factors may differ in their ability to bind closed *versus* open chromatin. Chromatin-immunoprecipitation of Hox factors followed by genomic sequencing (ChIP-seq) in *Drosophila* cell culture found that two Hox factors are largely constrained by nucleosome positioning [119]. In this study, ChIP was performed for transiently transfected Ubx or Abd-A in *Drosophila* Kc167 embryonic cell lines, which revealed that more than 94% of Ubx and Abd-A ChIP peaks occurred within pre-existing DNaseI hypersensitive regions of the genome. The findings for Ubx are consistent with the global accessibility profiles found in the haltere *vs*. wing described above [118]. In contrast to Ubx and Abd-A, however, a substantial proportion (25%) of Abd-B-associated ChIP peaks in *Drosophila* cell culture were located in previously closed chromatin [119]. These studies suggest that Hox factors may fundamentally differ in their ability to associate with nucleosome-bound genomic regions.

The finding that at least a subset of Hox factors bind predominantly open chromatin leads to an interesting question: how do Hox factors gain access to CRMs that exist in closed genomic states? At least part of the answer appears to be through interactions with PBC and HMP proteins. As described earlier, heterodimerization of PBC and HMP allows translocation of the PBC proteins from the cytoplasm to the nucleus [91]. Since Kc167 cells lack Hth expression, Exd remains cytoplasmic, and thus, the above ChIP-seq studies performed on Ubx and Abd-A were done in the absence of PBC and HMP proteins. To determine what impact Exd and Hth expression has on Hox binding profiles, the authors performed complimentary ChIP-seq studies in cells co-transfected with Hth [119]. These experiments revealed that the proportion of Ubx ChIP peaks in DNaseI insensitive regions expanded from 5% to 17%, and the total number of Ubx ChIP peaks doubled. Moreover, the additional sites that were bound by Ubx were enriched for PBC and HMP binding motifs. Altogether these studies suggest that by itself, Ubx does not substantially change nucleosome positioning; and that PBC and HMP proteins enhance Ubx binding to a greater number of sites, including those in closed chromatin.

Mechanistic support for the idea that PBC and HMP proteins can change the chromatin status of Hox targets prior to Hox binding comes from recent studies in zebrafish [120]. A complex of HoxB1b-Pbx-Prep/Meis activates *hoxb1a* transcription in the early zebrafish embryo [66]. Maternally-loaded Pbx and Prep bind the *hoxb1a* locus and cause histone acetylation of the locus prior to the expression of HoxB1b or transcription of *hoxb1a* [121]. Additionally, Pbx and Prep were shown to recruit RNA polymerase II to the *hoxb1a* promoter and maintain RNA polymerase II in a paused state. Slightly later in embryogenesis, HoxB1b is expressed and binds the *hoxb1a* locus, where it recruits factors that phosphorylate RNA polymerase II to promote transcriptional elongation [120]. Given the role of histone acetylation in changing nucleosome positioning, these studies suggest that PBC and HMP open chromatin and allow Hox factors to gain access to targets [122].

**Figure 4:**
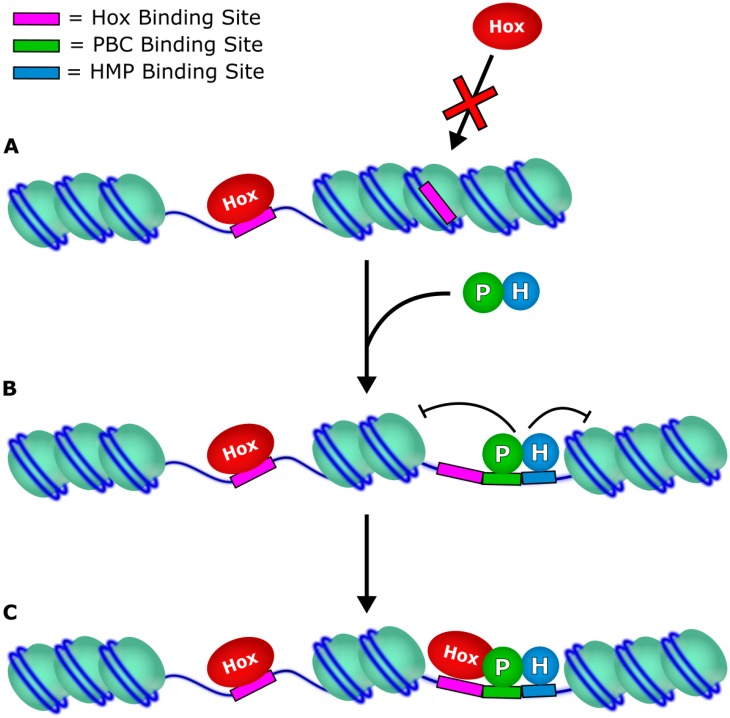
PBC and HMP can provide Hox factors access to targets. (**A**) Evidence suggests that Hox factors are typically limited to binding DNA in open chromatin regions [119]. (**B**,**C**) Expression of PBC and HMP protein has been shown to allow binding of Hox proteins to previously closed chromatin [119]. Prior to Hox binding, HMP and PBC proteins can bind DNA to promote chromatin modifications associated with chromatin opening, such as acetylation [120].

In total, these studies demonstrate that PBC and HMP proteins can contribute to Hox genomic binding patterns (Figure 4). Hence, PBC and HMP binding sites in Hox-regulated CRMs may provide Hox factors greater access to CRMs. Consistent with this idea, several genome-wide binding studies demonstrate substantial overlap between the binding of Hox factors and PBC/HMP proteins [123,124,125]. However, these studies did not determine if the PBC/HMP factors were bound to genomic regions prior to Hox factor recruitment *versus* direct cooperative binding of PBC/HMP/Hox complexes to DNA. Thus, future studies on the potential role of PBC/HMP proteins as pioneer factors are needed to determine if PBC/HMP proteins broadly define Hox target selection via the opening of chromatin prior to Hox factor recruitment.

## 4. Effect on Transcription

Like many TFs, individual Hox factors can both activate and repress target expression [38,126]. Although, Hox factors have been shown to recruit and/or interact with factors known to affect transcriptional outcomes, *i.e.*, chromatin remodelers, the mediator complex and the general transcriptional machinery [16,17,124,127,128,129,130,131], it is largely unclear what determines whether a Hox factor will act as an activator or a repressor on a given target gene. One example that explores the mechanism of Hox-mediated repression *versus* activation is the mammalian *osteocalcin* promoter [128,132,133]. The *osteocalcin* promoter contains adjacent Hox-Pbx binding sites, and in pre-osteoblasts, Pbx1 complexes with HoxA10 and recruits HDACs to the *osteocalcin* promoter, which represses gene expression. However, as pre-osteoblasts differentiate into osteoblasts, Pbx1 expression decreases, and HoxA10 then recruits CBP/p300 to acetylate *osteocalcin* and activate gene expression. These data suggest that the same PBC-Hox binding site can function in either gene activation or repression dependent on the presence of a PBC protein.

The binding of other TFs besides PBC and HMP can also influence whether a Hox factor activates or represses a target gene. For example, the Abd-A Hox factor can both activate the expression of *rhomboid* in abdominal sensory cells and repress the expression of Distal-less (Dll) in the abdominal ectoderm [40,46]. As described above, Abd-A activates the *rhoA* CRM via interactions with Pax2, and the RhoA CRM contains nearby binding sites for both of these factors [47]. However, Abd-A represses Dll expression via multiple Hox/PBC and Hox/HMP binding sites, and this repression activity requires the nearby binding sites for two additional transcription factors, Sloppy-paired (Slp, a FoxG homolog) and Engrailed (En), which are known to recruit the Groucho co-repressor protein [46,134,135,136]. Hence, the decision for this Hox factor to activate *versus* repress is dependent on which additional TF sites are located nearby. Since we know a great deal about how the Hox factors regulate Dll expression, we use this example as a case study below to highlight different mechanisms of Hox specificity.

## 5. Case Study: The Distal-less Conserved Regulatory Element

A number of the concepts underlying Hox specificity are represented in a single case study of how different Hox factors bind to and regulate the *DCRE*, a Hox-regulated CRM that controls *Distal-less* (*Dll*) expression in *Drosophila* embryos. *Dll* expression is essential for the specification of appendages, such as legs from the thorax [137]. Therefore, the viability of *Drosophila* depends on activating *Dll* expression in thoracic segments and repressing its expression in the abdomen. Several *Dll* CRMs have been identified, and the DMX is the best-characterized regulatory element that is active in the early leg precursor cells [46,70,138]. Initial studies suggested the DMX is composed of separable activation (DMEact) and repression elements (DCRE) [39,46]. More recent studies paint a more complex picture with the DMEact and the DCRE contributing to both thoracic activation and abdominal repression [70]. For the purpose of this review, we will focus on how the DCRE utilizes several different Hox/PBC and Hox/HMP sites, as well as additional TF binding sites to contribute to paralog-specific activation *versus* paralog-specific repression.

**Figure 5:**
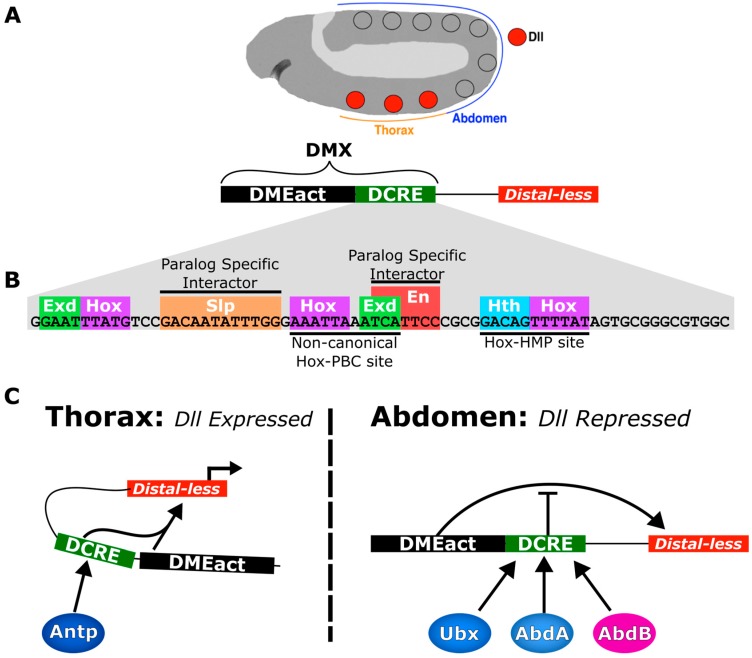
Regulation of *Distal-less (Dll*) gene expression by the *DCRE* in *Drosophila*. *Dll* is expressed in the *Drosophila* embryonic thorax, but not the abdomen. (**A**) *Dll* expression is controlled by two elements: the *DMEact* (an activator) and the *DCRE* (a mixed repressor and activator). *DMEact* is sufficient to drive expression in both the abdomen and the thorax, whereas the *DCRE* inhibits abdominal expression and enhances *DMEact* driven thoracic expression [70]; (**B**) Sequence of the *DCRE*, with known binding sites labeled. Slp and En binding sites are necessary for repressive *DCRE* activity in the abdomen [46,70]; (**C**) In the thorax, the chromatin structure of the *Dll* locus places the *DMEact* in proximity to the *Dll* promoter, whereas DNA looping between the DMEact regulatory region and the *Dll* promoter is not observed in abdominal segments [143]. These findings are consistent with results demonstrating that the DCRE “boosts” thoracic *DMEact* activation of *Dll* transcription via a mechanism dependent on Antp (the thoracic Hox factor) [70]. In contrast, the DCRE represses *Dll* transcription via mechanisms dependent on the abdominal Hox factors (Abd-A, Ubx and Abd-B) in the abdomen [46,70,138,139]. However, it is currently unclear if the thoracic and/or abdominal Hox factors directly regulate DNA looping between the distal leg enhancer and the proximal Dll promoter region.

Functional studies have revealed that the *DCRE* contains two Hox/PBC and one Hox/HMP site that are bound and regulated differentially by the Antp, Ubx, Abd-A and Abd-B Hox factors (Figure 5A,B) [46,70,138,139]. In the thorax, Antp cooperates with Exd/Hth to stimulate enhancer activity via binding the two Hox/PBC sites of the DCRE [70]. However, the Hox/HMP sites are not required for this activity, indicating that Antp may only specifically work on Hox/PBC sites and not Hox/HMP sites. While genetic and reporter studies have revealed that Antp is necessary for enhancing thoracic gene expression, it is currently unclear how Antp activates gene expression and whether additional binding sites in the DCRE are required for this activity.

In the abdomen, the abdominal Hox factors (Ubx, Abd-A and Abd-B) repress gene expression through the DCRE using two different cell-specific mechanisms. The *Drosophila* embryo is segmented, and each segment is composed of distinct anterior and posterior compartment cell types [140]. Mutagenesis studies on the DCRE revealed that mutations in the Hox/PBC and Hox/HMP sites resulted in a loss in repression activity in both cell types [46,70]. In contrast, a subset of mutations outside of the Hox/PBC and Hox/HMP sites revealed that distinct TF binding sites were required for anterior- *versus* posterior-compartment-mediated repression [46]. In anterior compartment cells, Slp binding sites are necessary to repress gene expression, whereas in posterior compartment cells, an En binding site is needed for repression [46]. Hence, both Slp and En are necessary for complete repression in the abdomen, because Slp expression is limited to the anterior compartment of embryonic segments, while En expression in limited to the posterior compartment of embryonic segments [141,142]. Slp and En both directly interact with Groucho, a histone deacetylase, and recruit it to CRMs [134,135,136]. Thus, it is thought that abdominal Hox factors promote a repressive chromatin state on the DCRE. Moreover, chromatin studies of the *Dll* locus demonstrate that in the thorax, the *DMEact* and *Dll* promoter physically communicate (*i.e*., DNA looping), but *DCRE*-mediated repression in the abdomen disrupts this communication [143] (Figure 4B). These data help to demonstrate how the same Hox-regulated CRM can mediate multiple outputs depending on the cellular context.

Our advanced understanding of how the DCRE mediates three distinct functions (thoracic activation *vs*. anterior and posterior compartment repression) raises multiple questions in regards to Hox specificity. First, since Slp and En are expressed in both the abdomen and the thorax, so why does Antp not repress gene expression via the *DCRE*? Different preferences for TF interactors provide insight into this question: abdominal Hox factors have been shown to form complexes with En on DNA and to interact with Slp via fluorescent complementation assays, but Antp fails to do so in the same assays [46,103]. Hence, without being able to interact with Slp an En, Antp is unable to repress gene expression via the *DCRE*.

Second, how do the specific sequences found within the two Hox/PBC and one Hox/HMP binding sites contribute to paralog specificity? As described above, the Hox/HMP site only contributes to abdominal repression and not to thoracic activation [70]. This finding is consistent with previous studies that suggest that only posterior Hox factors mediate strong interactions with HMP proteins on Hox/HMP sites [26,44,45]. In addition, the analysis of the two Hox/PBC sites suggests that both are non-optimal sequences with either an abnormal spacing (inclusion of an additional nucleotide between the PBC and Hox site) or with several mismatches from consensus PBC/Hox sites [23]. Moreover, Electromobility Shift Assays (EMSAs) demonstrate that Abd-A forms stronger complexes with Exd and Hth on the *DCRE* sequence than does Antp [70]. This may be due to the fact that the *DCRE* contains adjacent Hox-Hth binding sites that favor more posterior Hox factors [26,44,45]. Moreover, the molecular basis of this difference in affinity may be related to the presence of the UbdA motif in Abd-A (and Ubx), whereas Antp lacks this motif. For example, mutations in the UbdA motif in Ubx disrupt *DCRE* binding and repression more than mutations in the HX motif [86]. These findings suggest that the DCRE sequence achieves paralog specificity by favoring Hox paralogs that contain the UbdA motif (Ubx and Abd-A, not Antp).

Third, does the arrangement of Hox-PBC-HMP binding sites contribute to Hox paralog specificity? It has been previously suggested that the arrangement of Hox-PBC-HMP binding sites may contribute to the activity of Hox-regulated CRM independent of paralog specificity [144]. To test this idea, we recently altered the configuration of Hox sites within the DCRE by swapping them with the Exd/Hth/Hox site found within the RhoA CRM [70]. As described, Abd-A/Exd/Hth complexes repress gene expression via the *DCRE* and activate gene expression via the *RhoA*. We found that this hybrid CRM is able to mediate gene repression equivalently to wild-type *DCRE* in the anterior compartments of the abdomen. These results demonstrate that, at least in this case, the Hox-PBC-HMP binding site arrangement does not determine the activity of the CRM.

Fourth, does post-translational modifications of Hox factors alter their ability to regulate the DCRE? In *Drosophila*, Ubx inhibits limb development in the abdomen by repressing expression of *Dll* via the DCRE [46,138]. However, Ubx orthologs in other arthropods have been shown to lack this repression activity [145,146]. In fact, Ubx is expressed in cells that give rise to legs along the trunk in *Artemia* (brine shrimp) [147,148]. In contrast to *Drosophila* Ubx, *Artemia* Ubx can be phosphorylated by CKII at serine and threonine residues in its C-terminal region [106]. Mutating phosphorylation sites in *Artermia* Ubx results in an increased ability to repress *Dll* when misexpressed in *Drosophila* embryos. Moreover, mutating *Drosophila* Ubx to add phosphorylation sites to its C-terminal region reduces the ability of the mutant Ubx to repress *Dll* expression [108]. These studies suggest that an evolutionary divergence of legs along the trunk of *Artemia* and *Drosophila* may be in part due to divergence in the ability of Ubx to be phosphorylated in these two species.

Fifth, do all of the Hox factors that regulate Dll expression require the function of Exd and Hth? Exd and Hth cooperate with Antp, as well as Ubx/Abd-A to mediate gene activation and repression, respectively [46]. However, Exd and Hth have been found to antagonize Abd-B-mediated repression at the DCRE [139]. In fact, Abd-B represses Hth expression in the most posterior abdominal segments during embryogenesis. Since Hth is necessary for nuclear localization of Exd, the repression of Hth expression by Abd-B results in a PBC/HMP-free region. Hence, Abd-B creates an Exd/Hth-free area and represses the *DCRE* in an Exd/Hth independent manner [91,139].

## 6. Conclusions

Throughout this review, we have described multiple mechanisms by which Hox paralogs may differentiate their binding specificities and activities. These include: (1) latent specificity revealed by PBC proteins; (2) utilization of low affinity sites; (3) multiple interaction surfaces for PBC proteins; (4) preference of HMP proteins to cooperatively bind DNA with posterior Hox paralogs; (5) differential affinity for other TFs; and (6) differential regulation by post-translational modifiers (Figure 2). Additionally, we reviewed how interactions with other TFs regulate genomic binding patterns of Hox factors, as well as their effect on transcription. While we focused on Hox factors in this review, many of the same problems of specificity presented here apply to other families of transcription factors. However, it is unclear whether the different TF families primarily rely on similar strategies or distinct strategies to overcome these problems of specificity. Elucidating the molecular mechanisms of how TFs bind specific targets and produce specific regulatory outcomes will be an important step in our understanding of how eukaryotes produce robust and specific gene expression patterns throughout development.

## Abbreviations

The following abbreviations are used in this manuscript:

CRM: *cis*-regulatory module
Lab: Labial
Scr: Sex-combs Reduced
Antp: Antennapedia
Ubx: Ultrabithorax
Abd-A: Abdominal-A
Abd-B: Abdominal-B
TALE: Three Amino-acid Loop Extension
Exd: Extradenticle
Hth: Homothorax
